# Genome-Wide Epistatic Network Analyses of Semantic Fluency in Older Adults

**DOI:** 10.3390/ijms25105257

**Published:** 2024-05-11

**Authors:** Qihua Tan, Weilong Li, Marianne Nygaard, Ping An, Mary Feitosa, Mary K. Wojczynski, Joseph Zmuda, Konstantin Arbeev, Svetlana Ukraintseva, Anatoliy Yashin, Kaare Christensen, Jonas Mengel-From

**Affiliations:** 1 Epidemiology, Biostatistics and Biodemography, Department of Public Health, University of Southern Denmark, 5230 Odense, Denmark; weilonglicn@outlook.com (W.L.); mnygaard@health.sdu.dk (M.N.); kchristensen@health.sdu.dk (K.C.); jmengel-from@health.sdu.dk (J.M.-F.); 2 Unit of Human Genetics, Department of Clinical Research, University of Southern Denmark, 5230 Odense, Denmark; 3 Division of Statistical Genomics, Department of Genetics, Washington University School of Medicine, St. Louis, MO 63110, USA; anping@wustl.edu (P.A.); mfeitosa@wustl.edu (M.F.); mwojczynski@wustl.edu (M.K.W.); 4 Department of Epidemiology, University of Pittsburgh, Pittsburgh, PA 15261, USA; zmudaj@edc.pitt.edu; 5 Biodemography of Aging Research Unit, Social Science Research Institute, Duke University, Durham, NA 27708, USA; ka29@duke.edu (K.A.); svetlana.ukraintseva@duke.edu (S.U.); aiy@duke.edu (A.Y.)

**Keywords:** semantic fluency, elderly, GWAS, epistasis, LLFS

## Abstract

Semantic fluency impairment has been attributed to a wide range of neurocognitive and psychiatric conditions, especially in the older population. Moderate heritability estimates on semantic fluency were obtained from both twin and family-based studies suggesting genetic contributions to the observed variation across individuals. Currently, effort in identifying the genetic variants underlying the heritability estimates for this complex trait remains scarce. Using the semantic fluency scale and genome-wide SNP genotype data from the Long Life Family Study (LLFS), we performed a genome-wide association study (GWAS) and epistasis network analysis on semantic fluency in 2289 individuals aged over 60 years from the American LLFS cohorts and replicated the findings in 1129 individuals aged over 50 years from the Danish LLFS cohort. In the GWAS, two SNPs with genome-wide significance (rs3749683, *p* = 2.52 × 10^−8^; rs880179, *p* = 4.83 × 10^−8^) mapped to the *CMYAS* gene on chromosome 5 were detected. The epistasis network analysis identified five modules as significant (4.16 × 10^−5^ < *p* < 7.35 × 10^−3^), of which two were replicated (*p* < 3.10 × 10^−3^). These two modules revealed significant enrichment of tissue-specific gene expression in brain tissues and high enrichment of GWAS catalog traits, e.g., obesity-related traits, blood pressure, chronotype, sleep duration, and brain structure, that have been reported to associate with verbal performance in epidemiological studies. Our results suggest high tissue specificity of genetic regulation of gene expression in brain tissues with epistatic SNP networks functioning jointly in modifying individual verbal ability and cognitive performance.

## 1. Introduction

As a popular neuropsychological test, semantic fluency (also called category fluency or free listing) measures the ability to name items from a given category, e.g., animals, during a given time interval. Semantic fluency impairment may be attributed to a wide range of neurocognitive and psychiatric conditions including, among others, Alzheimer’s disease, depression, and schizophrenia. Epidemiological analyses showed that, although the effect of sex on semantic fluency has been controversial [1,2,3] perhaps due to methodological issues [4], consistent influences by age and education have been reported with a negative effect of age, especially in late life, and a positive effect of education. To tease out the genetic and environmental components in the individual variation of semantic fluency, a recent multi-cohort twin study estimated a moderate heritability (h^2^ < 0.5), which was not modulated by age and education [2]. A moderate genetic contribution to semantic verbal fluency (h^2^ = 0.32) was also reported in a family-based study [5].

Despite the significant genetic background, efforts to identify the underlying genetic variants that contribute to semantic fluency have been very limited. As an early effort, Krug et al. [6] tested two single-nucleotide polymorphisms (SNPs), rs3918342 and rs1421292, in the D-amino acid oxidase activator gene (*G72)*, a gene which has been found to be associated with several psychiatric disorders, and found a significant correlation between rs1421292 polymorphism and semantic verbal fluency. In another candidate gene approach, Nicodemus et al. [7] analyzed 39 coding SNPs in candidate genes reported to associate with language and speech. A significant association with verbal fluency was observed for only one SNP, rs12133766 in the disrupted-in-schizophrenia-1 gene (*DISC1*). Currently, only one genome-wide association study (GWAS) on semantic fluency has been reported [8]. Despite a relatively large sample size, this family-based GWAS detected only one significant SNP, rs72687454, in the regulating synaptic membrane exocytosis 1 gene (*RIMS1*) (*p* = 4.7 × 10^−8^). The situation could imply that the genetic architecture of verbal ability is highly polygenic with each causative SNP constituting only a small fraction of the contributing factors, but that the epistatic interaction between SNPs may contribute to a larger extent [9]. The detection of SNPs with minor effects requires large sample sizes to obtain sufficient statistical power. One efficient approach to overcome the concern of statistical power is by performing network-based analysis that takes epistasis, i.e., interaction between SNPs, into account. The network-based analysis is also biologically important, as functional dependencies between genes are a defining characteristic of gene networks underlying quantitative traits [10]. 

Using a large collection of genome SNP genotype data from individuals enrolled in the Long Life Family Study (LLFS) [11], we performed a GWAS on semantic fluency in elderly individuals aged over 60 years to identify and assess SNPs of potential significance using a conventional GWAS pipeline. Next, we conducted a network-based analysis of the GWAS SNPs to construct and test SNP clusters or modules associated with semantic fluency using the weighted interaction SNP hub (WISH) network method [12]. The large collection of samples allowed partitioning the samples into a discovery and a replication set for replication and validation of our findings. 

## 2. Results

### 2.1. GWAS on Discovery Sample

**Figure 1:**
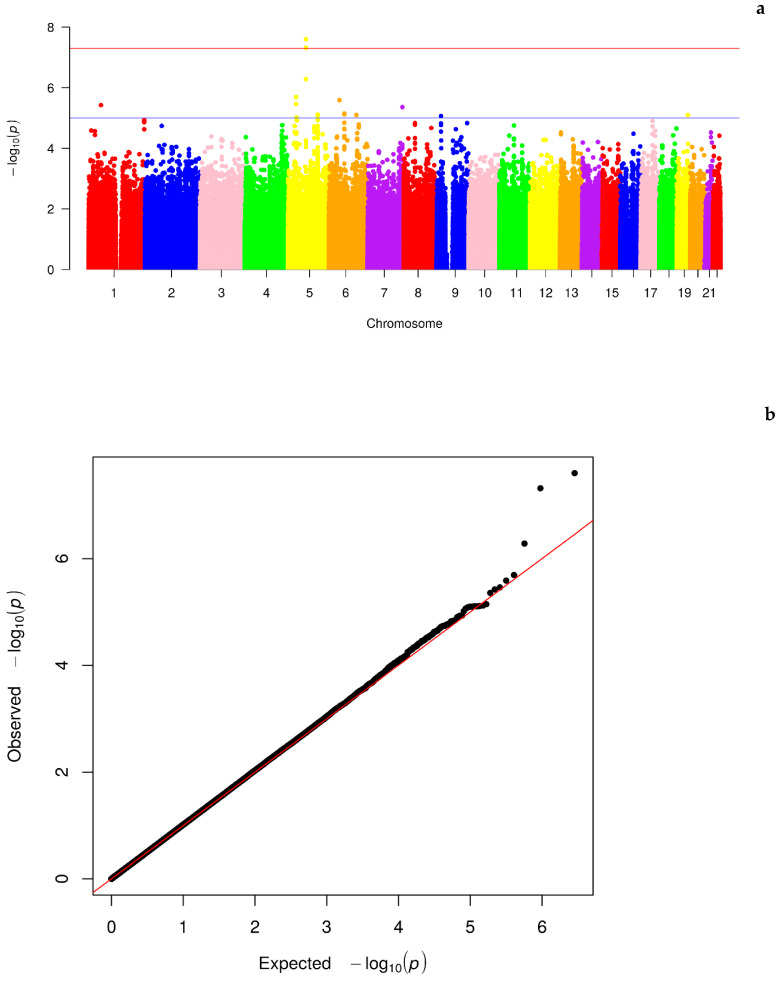
Manhattan (**a**) with red and blue lines indicating genome-wide and suggestive significances respectively, and QQ plot (**b**) of the GWAS on discovery sample. The top significant SNPs are located in the same region on chromosome 5, deviating significantly from the random diagonal line.

As the first step, we performed GWAS based on the genotyped SNPs from the American LLFS participants. After preprocessing and quality control, a total of 1,422,288 SNPs were available for testing. In the GWAS, we detected 2 SNPs that reached genome-wide significance (rs3749683, *p* = 2.52 × 10^−8^; rs880179, *p* = 4.83 × 10^−8^) and 16 SNPs with suggestive significance (5.21 × 10^−7^ < *p* < 9.65 × 10^−6^) (Table 1). Detailed statistics for the 74,270 SNPs with *p* < 0.05 can be found in Supplementary Table S1. Figure 1 displays the Manhattan plot (Figure 1a) and QQ plot (Figure 1b) for the GWAS results. The QQ plot shows no sign of inflation of statistical significance, indicating that GMMAT efficiently controlled for relatedness in the pedigree structure in making statistical inference. Three SNPs deviate from the random distribution of the diagonal line in Figure 1b; the two genome-wide significant SNPs mentioned above (intronic SNPs) and an additional SNP rs16877206, which are all located on chromosome 5 (Figure 1a). Moreover, most of the top SNPs have a low maf, with a median of 0.083 for SNPs with *p* < 1 × 10^−5^ (Supplementary Table S1). 

### 2.2. Analysis of Epistatic Networks

**Figure 2:**
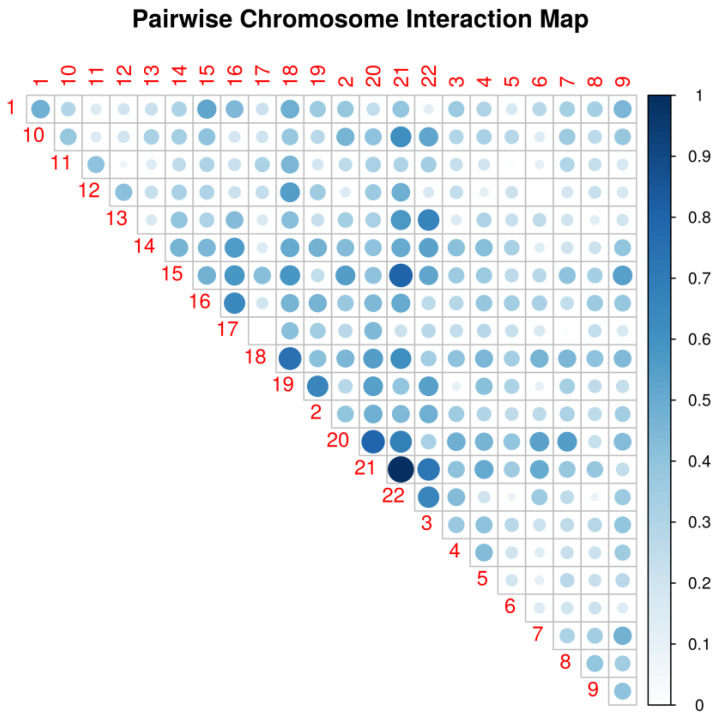
Pairwise chromosome interaction in association with semantic fluency. The most intensive SNP–SNP interaction is observed between chromosomes 21 and 22.

**Figure 3:**
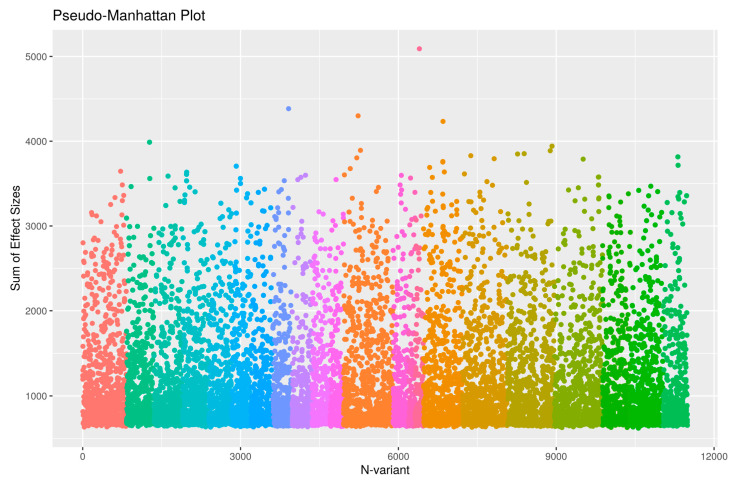
Pseudo Manhattan plot displaying the sum of effect size (sum over the −log likelihoods) of all interactions for each SNP across the genome for each of the 10,270 SNPs arranged by chromosome from chromosomes 1 to 22 using different colors.

Before construction and testing of the epistatic networks, we first filtered SNPs according to their GWAS *p* value by selecting 13,587 SNPs with *p* < 0.01 in accordance with the number of SNPs suggested by the authors of WISH (10,000 to 20,000 SNPs). Following the protocol steps suggested by the authors (see Section 4), we calculated the epistatic interactions based on semantic fluency and display the chromosomal hotspots of epistatic interaction in Figure 2. The figure shows that the pairwise SNP interaction is most evident in chromosomes 21 and 22, followed by 20 and 21, 21 and 15, etc. The LD pruning identified and removed 3317 SNPs in LD with tagging SNPs, leaving 10,270 SNPs for genome-wide epistatic analysis. Figure 3 displays a pseudo-Manhattan plot exhibiting the sum of effect sizes, which is the sum over the -log likelihoods of all interactions for each SNP across the genome, plotted for the 10,270 SNPs arranged by chromosome (differentially colored for chromosomes 1 to 22). It can be seen from the figure that many of the SNPs are highly interactive in modulating an individual’s measurement of semantic fluency. 

Based on SNP–SNP interaction patterns, the SNPs were clustered into 25 modules labelled using color names (Supplementary Table S2). In consideration of multiple testing, Table 2 presents only the top 5 modules with a *p* value below 0.01. Module Yellow (consisting of 951 SNPs) is most significantly associated with semantic fluency with a *p* value of 4.16 × 10^−5^, followed by module Turquoise (2085 SNPs) with *p* = 8.88 × 10^−4^, module Black (710 SNPs) with *p* = 2.24 × 10^−3^, module Blue (1115 SNPs) with *p* = 6.54 × 10^−3^, and a small module, Dark Gray (90 SNPs) with *p* = 7.35 × 10^−3^. 

### 2.3. Replication

For replication purposes, we first conducted a GWAS on the Danish LLFS cohort, which identified no genome-wide significant SNPs but 20 SNPs with suggestive significance. Based on the distribution of the GWAS statistics, we were able to test the enrichment of all the SNPs in each module in Table 2 for their association with semantic fluency. The enrichment analysis was performed using the gene-set test. Two modules were successfully replicated with *p* = 9.99 × 10^−5^ for module Turquoise, *p* = 3.10 × 10^−3^ for module Blue. One module, module Yellow was replicated with *p* = 0.067. The two smallest modules (Black and Dark Gray) were not significantly replicated.

### 2.4. Functional Interpretations

For the two significantly replicated modules, we moved on with functional annotations using relevant functions provided by VEGAS2 and FUMA. SNPs in each module were first mapped to genes and the statistical significance of each mapped gene was tested to find a list of genes with *p* < 0.01. The 2085 SNPs in the Turquoise module were mapped to 473 genes (Supplementary Table S3) among which 7 genes (*CSF2, IL3, DPP6, FRMD4A, SORCS2, ACSL6*, and *P4HA2-AS1*) were mapped with *p* < 1 × 10^−5^. The 1115 SNPs in the Blue module were mapped to 258 genes (Supplementary Table S4) and among them, 3 genes (*ARHGEF10, TRPM3*, and *LRP1B*) were detected with *p* < 1 × 10^−5^. Interestingly, for most of the top significant genes, their *p* values were lower than the *p* values of the most significant SNPs they carried, implying the enriched power by gene-based testing. 

**Figure 4:**
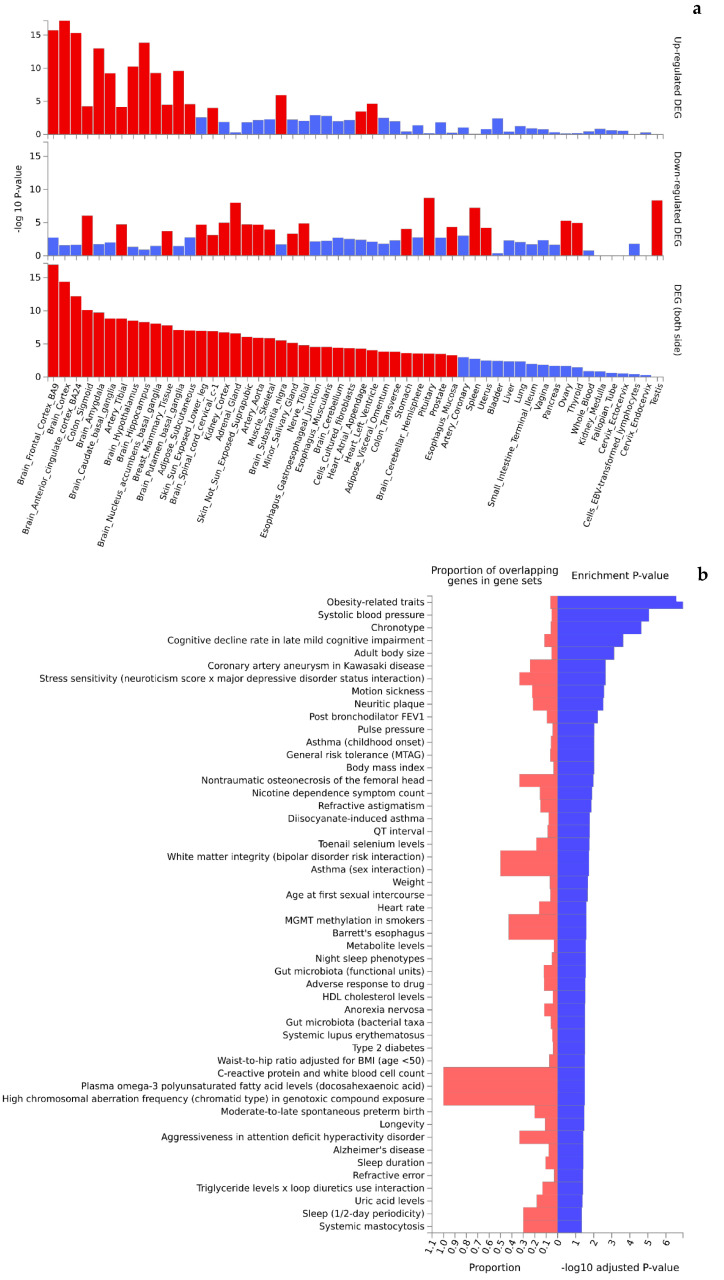
Significant enrichment (red colored) of tissue-specific gene expression (**a**) and GWAS catalog traits (**b**) by genes mapped to the Turquoise module.

**Figure 5:**
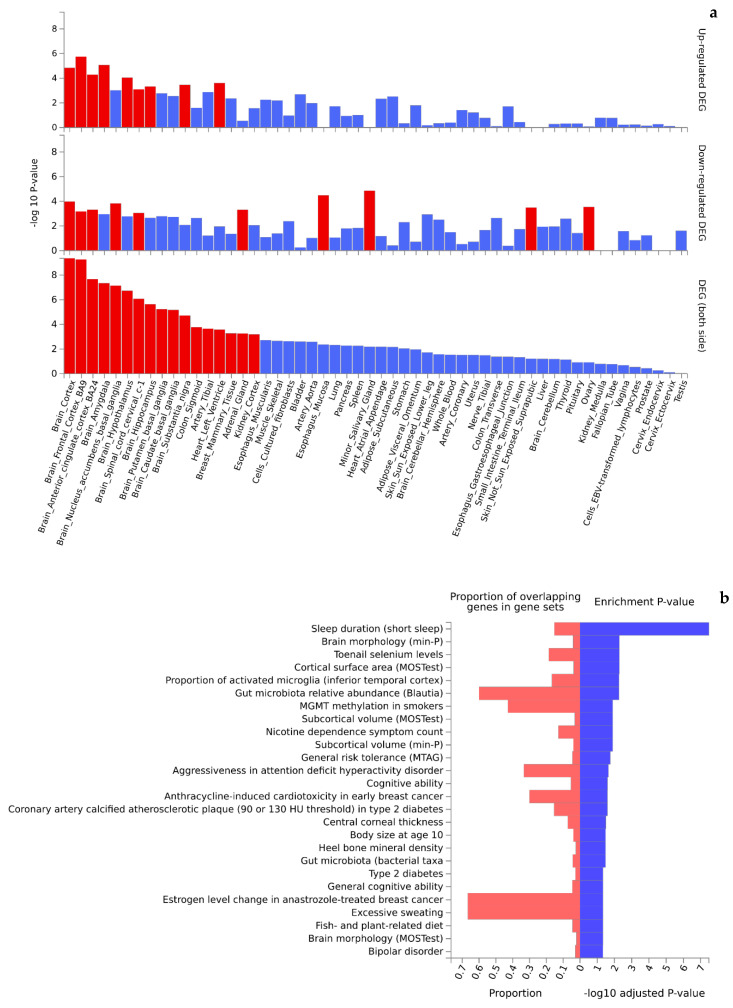
Significant enrichment (red colored) of tissue-specific gene expression (**a**) and GWAS catalog traits (**b**) by genes mapped to the Blue module.

Functional interpretation of the 473 Turquoise module genes revealed significant enrichment in up- and downregulated gene expression patterns in multiple tissues (Figure 4a). Of the 54 tissue types included in the GTEx v8 data, 36 were found to be differentially expressed (P_Bonferroni_ < 0.05) by genotypes of the Turquoise module genes. Interestingly, the top significant differentially expressed tissues (mainly upregulated gene expression patterns) were dominated by brain tissues (e.g., cortex, amygdala, basal ganglia, hypothalamus, and hippocampus) that are highly relevant to cognition. Further, the Turquoise module genes were significantly enriched in 36 of the 50 GWAS catalog traits (adjusted *p*-value < 0.05) (Figure 4b, Supplementary Table S5) topped by obesity-related traits (*p* = 2.57 × 10^−11^), systolic blood pressure (*p* = 3.95 × 10^−9^), chronotype (*p* = 1.57 × 10^−8^), cognitive decline rate in late mild cognitive impairment (2.10 × 10^−7^), and adult body size (*p* = 8.42 × 10^−7^). Likewise, functional interpretation of the 258 Blue module genes also identified significant enrichment (P_Bonferroni_ < 0.05) in up- and downregulated gene expression patterns by tissue types (Figure 5a). Similar to the Turquoise module, the top significant tissues were again dominated by brain tissues. Analysis of traits in the GWAS catalog identified 26 traits over-represented by the genes of the Blue module (Figure 5b, Supplementary Table S6) topped by sleep duration (short sleep) (*p* = 7.49 × 10^−12^), brain morphology (min-*p*) (*p* = 3.88 × 10^−6^), toenail selenium levels (*p* = 4.17 × 10^−6^), and cortical surface area (multivariate omnibus statistic test, MOSTest) (*p* = 4.70 × 10^−6^).

## 3. Discussion

Using the high-resolution genome-wide SNP data available for participants in the Long Life Family Study, we performed a GWAS- and a network-based epistatic association study to identify muti-locus SNP–SNP interaction effects that contribute to the observed individual variation in semantic fluency. As shown in Figure 3, SNPs are frequently highly interactive across the genome in making their contributions to verbal fluency, an important phenomenon that has rarely been considered in conventional GWASs. Results from our network analysis indicate that the epistasis approach not only improves the statistical power of genome-wide association analysis, but also helps to discover biologically meaningful findings to enrich our understanding of the genetics of verbal fluency performance. 

In the GWAS performed using the discovery sample, two SNPs, rs3749683 and rs880179, were detected as having genome-wide significance. Both SNPs are positioned in or near *CMYA5* (rs3749683 is an intron variant, and rs880179 a 500B downstream variant) on chromosome 5, a gene that confers risk for schizophrenia and major depressive disorder [13,14] and cardiomyopathy [15,16]. Although our major interest is in genome-wide epistasis analysis, the limited number of significant findings on a single SNP (gene) level already indicates potential genetic overlap between verbal fluency and other complex neuropathogenic mechanisms. Of course, this point is more clearly illustrated by the functional interpretations of the significant modules or networks identified in the network-based analysis.

The top two significant genes of the Turquoise module, *CSF2* and *IL3*, are both cytokine genes that mediate cell–cell communication in the immune system. A recent study reported that *CSF2* activity is significantly associated with memory and processing speed [17]. The study also found that plasma immune markers have an independent association with cognition beyond what is due to traditional risk factors for cognition. Multiple studies have consistently shown the involvement of *IL3* signaling in the pathophysiology of schizophrenia, among which Xiu et al. [18] found that IL3 may be involved in the immediate memory deficits in the chronic phase of schizophrenia. Another top significant gene, *DPP6*, is expressed in multiple regions of the brain and has been found to be multifunctional with an additional, independent role in synapse formation and maintenance [19]. Among the top significant genes of the Blue module, *TRPM3* and *LRP1B* are receptor genes involved in multiple functions such as cell activation, and cell adhesion and signaling pathways. *TRPM3* has been related to neurodevelopmental disorders [20] concerning speech/language skills and mild-to-severe intellectual disability, while the *LRP1B* gene was found to be a major risk factor in the progression to Parkinson’s disease dementia [21]. These observations on the top genes could imply different functional profiles of the two modules in modulating semantic verbal fluency through diverse pathways.

The GWAS catalog traits significantly enriched by genes of the Turquoise module are topped by obesity and systolic blood pressure. Metabolic risk factors, hypertension, and diabetes, among others, have been hypothesized to play an important role in the pathogenesis of Alzheimer’s disease and the development of vascular dementia. Specifically, a recent study found a significant difference in performance between patients with metabolic syndrome and controls, both in the phonetic (*p* < 0.01) and semantic fluency trials (*p* < 0.001) [22]. For the third enriched GWAS catalog trait, chronotype, a recent study found that in later adulthood, those who habitually get up early have better verbal skills [23]. Similar observations have been reported by Hidalgo et al. [24] and Heimola et al. [25]. As sleeping patterns have been related to obesity [26], the role of chronotype in verbal processing can be complex or perhaps indirect. What is important here is that the reported correlations between these traits and verbal performance are genetically modulated.

Sleep duration (short sleep) is the trait most significantly enriched by the Blue module genes. In a large-scale twin study, Vo et al. [27] recently reported a large genetic influence on semantic fluency and episodic memory at shorter sleep durations. Interestingly, the SNPs and their mapped genes of the Blue module provide a molecular genetic architecture to the estimated genetic contribution from the twin study. Among the other top GWAS catalog traits significantly enriched by the Blue module, brain morphology, cortical surface area, and subcortical volume are all structural features of the brain, which have been associated with verbal fluency in developing children [28,29]. Another brain-related significant trait is the proportion of activated microglia (inferior temporal cortex) (Figure 5b). It has been shown that microglial activation is already present before the onset of dementia in populations at genetic risk of Alzheimer’s disease [30], and brains resilient to Alzheimer’s disease display decreased microglia and astroglia activation [31]. Overall, the top GWAS catalog traits enriched by the Blue module suggest that the module represents interactive genetic variations that influence both structural and functional changes of the human brain in relation to verbal processing and cognition. Other interesting traits include toenail selenium levels and gut microbiota relative abundance, which are also reported to associate with verbal fluency [32] and cognitive impairment [33], again suggesting a high functional relevance of the Blue module to verbal ability. 

Finally, the top significantly enriched tissue types by both the Turquoise and the Blue modules are all dominated by genes expressed in brain tissues, e.g., cortex, amygdala, hippocampus, and basal ganglia (Figure 4a and Figure 5a). While these results imply involvement of gene activity in these tissue types with verbal ability, more importantly, the results suggest tissue specificity of genetic regulation of gene expression [34] where SNPs in the significant modules could serve as expression quantitative trait loci (eQTLs; cis-eQTLs or trans-eQTLs) that regulate jointly the expression pattern of multiple genes in modifying individual verbal ability and cognitive performance. Identifying and characterizing the complex eQTL networks call for more efforts in computational bioinformatics and multi-omics analysis. 

## 4. Materials and Methods

### 4.1. The Long Life Family Study

The LLFS is a multicenter family-based study of healthy aging and longevity with families recruited by four study centers in New York, Boston, and Pittsburgh in the United States, and in Denmark. Detailed description of eligibility criteria can be found elsewhere [35]. A total of 539 pedigrees consisting of 4953 individuals were recruited. This study included 2289 individuals with an age over 60 years (median age 81; 1086 males, 1203 females; 463 families) from the three American centers as the discovery sample and 1129 individuals aged >50 years (median age 65; 524 males, 605 females; 76 families) from Denmark for replication analysis (Table 3). The division of discovery and replication samples took into account geographical location of participants to ensure complete independence and reasonable sample sizes. The study approvals were obtained from the institutional review boards at each participating institution with informed consent obtained from all participants.

### 4.2. Semantic Fluency Measurement

The semantic or category fluency was measured by the number of animals named in 60 s as the total score. The median score for the discovery sample was 17 (range: 0–45) and for the replication sample 21 (range: 1–43). No significant difference in the total score was observed between the discovery and replication samples (*t*-test statistic 0.024, *p* value 0.98). Before statistical analysis, we applied the rank-based inverse normal transformation (INT) to the fluency measurements to counteract departures from normality [36]. INT first maps the sample measurements onto a probability scale using the empirical cumulative distribution function where the observed values are replaced with fractional ranks, then transforms the observations into Z-scores using the probit function. Currently INT is one of the most popular approaches to achieve normally distributed traits (or normally distributed residuals) in genetic association studies [37,38,39].

### 4.3. Genome-Wide SNP Genotyping, Preprocessing, and Quality Control

Genome-wide SNP genotype data were generated using the Illumina Omni2.5 SNP array, a high-density array covering 2.5 million SNPs in the human genome. Quality control was performed at the data coordinating center (Washington University, St. Louis) and standard procedures were applied. A total of 1,901,928 SNPs were genotyped in the discovery sample. Among them, 476,614 SNPs had minor allele frequency (maf < 0.01) and were removed from subsequent analyses. The remaining SNPs were tested for Hardy–Weinberg equilibrium (HWE) and we further dropped 3026 SNPs with *p* < 1 × 10^−6^ in the HWE testing. In the network analysis, SNPs were also filtered based on linkage disequilibrium (LD) measures between a pair of SNPs within a block of SNPs sorted by chromosomal coordinates and showing high LD (D’ or r^2^ ≥ 0.9), the LD blocks. 

### 4.4. GWAS Statistical Analysis

Considering the pedigree structure in the LLFS SNP data, association with the INT-transformed fluency levels by individual SNPs was tested using the generalized linear mixed model (GLMM) association tests implemented in the R package GMMAT. GMMAT fits GLMMs with covariate adjustments (here age and sex) and random effects to account for population structure and family relatedness and performs score tests for each genetic variant [40,41]. The R package GEMMA [42] was used to compute a genetic relationship matrix (GRM, an empirical kinship matrix) to account for the covariance structure of genetic relatedness in the LLFS samples, which is included in the fitting of GLMMs by GMMAT. Genome-wide significance of SNPs was defined as *p* < 5 × 10^−8^, with *p* < 1 × 10^−5^ indicating suggestive significance. 

### 4.5. Epistatic Network Analysis

The genome-wide epistatic network analysis was performed by applying the WISH-R package (version 1.0) [12] using the weighted interaction SNP hub (WISH) network method [43]. The main idea behind network analysis is to avoid the stringent thresholds for genome-wide significance at a single SNP level in conventional GWAS, which lead to loss of biologically relevant but statistically insignificant SNPs [44]. WISH is developed to capture SNPs of marginally significant small effects but manifest biologically meaningful and significant interactions with other SNPs. 

Analysis of SNP-SNP interaction: The method first reduces dimensionality of the interactive SNPs by filtering SNPs based on their GWAS *p* values using a desired but loose cutoff (here *p* < 0.01). The selected SNPs are pruned for linkage disequilibrium (LD) by creating blocks of input SNP genotypes based on LD (sorted by genomic coordinates and chromosome) and selecting tagging variants in each block, with a maximum block size of 1000, and threshold of D’ ≥ 0.9. Then, a matrix of epistatic correlation between all pairs of remaining SNPs is established. 

The following linear model is used for estimating interaction between two SNPs:

$$y=\mu +\beta_{i}{SNP}_{i}+\beta_{j}{SNP}_{j}+\beta_{ij}({SNP}_{i}\times {SNP}_{j})+\varepsilon$$

where *y* is the phenotype of interest (here transformed fluency level), *μ* is the intercept, *β_i_* and *β_i_* are the main effects of SNPs *i* and *j*, and *β_ij_* represents the epistasis of the two loci. *ε* is the random residual effect. The genotypes of *SNP_i_* and *SNP_j_* are coded as 2 (homozygote minor allele), 1 (heterozygote) or 0 (homozygote major allele). The estimated epistatic interactions (*β_ij_*) can be visualized by the quantile values of the significance of the interaction between chromosomes with a quantile size of 0.9. Visualization of the chromosome pairwise relative strength of epistatic interaction ranges from 1 (strongest) to 0 (weakest). It indicates the chromosomal hotspots for the interaction for measured fluency levels. 

Epistatic network construction and association analysis: The construction of genomic interaction networks or modules is based on the WGCNA framework [45] using the matrix of epistatic interactions between all pairs of filtered SNPs. This step performs hierarchical clustering, SNP selection, and parameter selection for module construction. Thereafter, association of each constructed module with semantic fluency is assessed by calculating SNP module eigengene (ME) and fitting GLMMs adjusting for age, sex, and genetic relatedness using GMMAT. Similar to the GWAS statistics, the fitting of GLMMs includes a GRM estimated by GEMMA to account for genetic correlation in the sample. The SNPs from the significant modules were termed as hub-SNPs and selected for further analysis.

### 4.6. Replication Strategy

The identified significant modules or SNP networks were replicated for their association with semantic fluency in the independent replication sample of Danish LLFS participants (1129 individuals). We first performed a GWAS on the Danish sample using the same procedure and setup as for the discovery GWAS on the American LLFS participants. Then, for each module (including all SNPs in the module), we assessed its overall association with fluency measurement using the geneSetTest() of the R package limma [46]. The function tests whether a set of SNPs is highly ranked relative to other SNPs in terms of a given statistic (here, the score statistic from GMMAT) from the GWAS on Danish LLFS participants. The function allows specifying the alternative hypothesis as one-sided (positive or negative association), two-sided (either positive or negative associations), and mixed (regardless of direction of association). Considering multiple testing, we used a stringent threshold of *p* < 0.01 for the enrichment of the module SNPs in association with semantic fluency in the replication sample to define a successful replication. 

### 4.7. Functional Annotation of Modules

Functional annotation of SNPs in a significantly replicated module was achieved using VEGAS2 [47] for gene-based testing and FUMA (functional mapping and annotation of GWAS results, https://fuma.ctglab.nl, accessed on 1 February 2024), a platform developed to annotate, prioritize, visualize, and interpret GWAS results [48]. VEGAS2 maps SNPs of a module to genes if SNPs are within 50 kb of the 5′ and 3′ UTR of a gene (build hg19/GRCh37). The mapped genes are then tested for statistical significance by first converting the n SNPs’ *p*-values to upper tail χ2 statistics with one degree of freedom (df) and then summing up to calculate a gene-based test statistic that would have a χ2 distribution with n degrees of freedom under the null hypothesis [47]. Significant genes (*p* < 0.01) are forwarded to FUMA to obtain insight into putative biological mechanisms of input genes using the GENE2FUNC function. Here, a competitive approach is used to test whether the genes of a functional category (traits based on the GWAS catalog and tissue types based on GTEx v8 RNA-seq data) are more strongly associated with semantic fluency level than other genes using the hypergeometric test. 

## Figures and Tables

**Table 1:** GWAS result for top SNPs with *p* < 1 × 10^−5^.

SNP	SCORE	*p* Value	Chromosome	Position	MAF	Gene
rs3749683	−114.004	2.52 × 10^−8^	5	79095145	0.071977	*CMYA5*
rs880179	−110.539	4.83 × 10^−8^	5	79096053	0.071383	*CMYA5*
rs16877206	−108.272	5.21 × 10^−7^	5	79091514	0.080384	*CMYA5*
rs16902350	−104.304	2.03 × 10^−6^	5	35625482	0.061807	*SPEF2*
rs76918654	−48.855	2.58 × 10^−6^	6	46970372	0.01662	*ADGRF1*
rs57516403	−101.665	3.47 × 10^−6^	5	35625299	0.061793	*SPEF2*
rs55668426	163.768	3.78 × 10^−6^	1	54952406	0.227273	
rs12539925	−146.186	4.39 × 10^−6^	7	153561830	0.17605	*DPP6*
rs72881480	−103.693	7.16 × 10^−6^	6	68202554	0.08081	
rs113296667	−103.77	7.60 × 10^−6^	6	68232235	0.08312	
rs3916441	190.202	7.71 × 10^−6^	5	131369241	0.484112	
rs3763115	189.981	7.83 × 10^−6^	5	131364181	0.48029	
rs6596051	189.981	7.83 × 10^−6^	5	131363937	0.48029	
rs75336718	−81.2759	7.96 × 10^−6^	6	121095574	0.057109	
rs62131031	132.735	7.99 × 10^−6^	19	48705354	0.158641	*CARD8*
rs4705841	189.733	8.21 × 10^−6^	5	131364510	0.482208	
rs10811051	−183.463	8.65 × 10^−6^	9	18861021	0.457587	*SAXO1, ADAMTSL1*
rs6869247	−146.101	9.65 × 10^−6^	5	37986588	0.197251	

**Table 2:** Top 5 significant modules or networks detected with *p* < 0.01.

Module Names	Module Size (Number of SNPs)	Module-Trait Association (Coefficient)	*p* Value
Yellow	951	−4.604	4.16 × 10^−5^
Turquoise	2085	−3.384	8.88 × 10^−4^
Black	710	−3.216	2.24 × 10^−3^
Blue	1115	−2.445	6.54 × 10^−3^
Dark Gray	90	−2.256	7.35 × 10^−3^

**Table 3:** Descriptive statistics of samples.

	Discovery Sample	Replication Sample
Sample size	2289	1129
Age		
Median	81	65
Range	61–110	51–104
Sex		
Male	1086	524
Female	1203	605
Semantic fluency		
Median	17	21
Range	0–45	1–43

## Data Availability

The original contributions presented in the study are included in the article/Supplementary Materials, further inquiries can be directed to the corresponding author/s.

## Supplementary Materials

The supporting information can be downloaded at: https://www.mdpi.com/article/10.3390/ijms25105257/s1.
